# Review on "High Performance Computing in Science and Engineering, Munich 2004" edited by S. Wagner, W. Hanke, A. Bode and F. Durst

**DOI:** 10.1186/1475-925X-4-26

**Published:** 2005-04-12

**Authors:** Kurt Saetzler

**Affiliations:** 1 Bioinformatics Research Group, School of Biomedical Sciences, University of Ulster, Coleraine, Co. Londonderry, BT52 1SA, Northern Ireland, UK

The rather general nature of the book title raises many expectations with respect to High Performance Computing (HPC). One is tempted to believe that this book discusses in greater detail the different HPC architectures, their advantages and disadvantages, shows different implementation strategies to make use of the enormous computing power offered by these architectures, lists software packages and libraries helping in parallelizing code, gives tips for benchmarking, talks about compiler issues, batch processing, addresses shared and distributed memory models and overheads associated with inter-process communication. The summary on the back of the book, which states that authors from "leading-edge research groups in the field of scientific computing present their outstanding projects" and "describe their scientific background, their resources requirements with respect to top-level supercomputers, and their methods for efficient utilization of the costly high-performance computing power" just adds to this expectation.

Unwrapping the book from its lamination soon reveals that one should have paid more attention to the part 'Munich 2004' within the title. Reading the first pages of the book and the preface shows that the book is a collection of selected status reports that were presented at a workshop in Munich on the 2^nd ^and 3^rd ^of March 2004. The participants of the workshop were all members of the Competence Network for Technical/Scientific High Performance Computing (KONWIHR) working closely together with the Centre for High Performance Computing in Bavaria (HLRB). Although the editors stress the ongoing international collaborations from several research groups within the network, the chosen framework for the book drastically narrows the view on HPC to the KONWIHR perspective. It seems that publishing an official Springer book was much more appealing to the editors than just submitting an internal technical report to the sponsors of KONWIHR, such as the German Research Foundation (DFG) and the Bavarian State Ministry of Science, Research and Arts.

Unfortunately, the editors did not make an increased effort to make this a book that focuses on HPC. The articles presented to the reader are highly variable in quality and only a minority meets the expectations raised on HPC. Some articles have not been thoroughly proofread and still contain many typing errors. Some figure legends were not translated into English. The motivation for using HPC was poor and often given as *need for speed*, but the gain in speed was rarely detailed. Most importantly, the vast majority of articles focus on very specific applications, which require a substantial degree of background knowledge in that particular field. As the book covers "modelling and simulation in the disciplines Biosciences, Chemistry, Chemical Physics, Solid-State Physics, High-Energy Physics, Astrophysics, Geophysics, Computational Fluid Dynamics, and Computer Science" it is impossible to understand every article in greater detail. And finally, although the number of disciplines covered is vast, the book does not give a comprehensive overview on HPC driven research looking at each discipline separately, which is particularly true for the Biosciences.

I personally think that the book title should have been chosen with more care clearly reflecting the nature of this book. As many of the presented articles seem to be partly published elsewhere, an electronic version of the collection of reports would have been a more efficient way in disseminating the results of the KONWIHR project. This would have had the additional advantage that the articles could have been electronically searched for specific key words. The book is indeed not suited for somebody generally interested in HPC, but it might be a good point of reference for somebody working on a very similar project than the ones covered by the articles.

